# Tumour-associated macrophages in human meningiomas

**DOI:** 10.1371/journal.pone.0319960

**Published:** 2025-05-13

**Authors:** Rahmina Meta, Henrik Sahlin Pettersen, Sofie Eline Tollefsen, Borgny Ytterhus, Øyvind Olav Salvesen, Wenche Sjursen, Sverre Helge Torp

**Affiliations:** 1 Department of Clinical and Molecular Medicine, Faculty of Medicine and Health Sciences, Norwegian University of Science and Technology (NTNU), Trondheim, Norway; 2 Department of Pathology, St. Olavs Hospital, Trondheim, Norway; 3 Department of Public Health and Nursing, Faculty of Medicine and Health Sciences, Norwegian University of Science and Technology (NTNU), Trondheim, Norway; 4 Department of Medical Genetics, St. Olavs Hospital, Trondheim, Norway; PLOS: Public Library of Science, UNITED STATES OF AMERICA

## Abstract

Tumour-associated macrophages (TAMs) are regarded as potential therapeutic targets due to “pro-tumoral” and “anti-tumoral” phenotypes. Human meningiomas contain considerable number of TAMs, but their clinical impact is sparsely investigated in these tumours. The aim of this study was therefore to investigate the presence, morphology, and distribution of TAMs in human meningiomas, and relate these findings to histopathology, meningioma subtypes, World Health Organization (WHO) grade, risk of recurrence, and overall survival. In this study, 147 WHO grade 1 and 2 primary meningiomas prepared as tissue micro arrays were included. Standard immunohistochemistry, with the antibodies Iba1 as a pan-marker for “all TAMs”, iNOS for M1 and Arginase 1 for M2 TAMs, was performed to investigate their infiltration in the meningioma tissue. The immunostainings were scanned and analysed digitally. TAMs were found in most of the meningiomas with varying amount of ramified and amoeboid appearances. The quantity of total TAMs (Iba1-stained) was found to be significantly higher in the age group ≥ 60 years compared with the younger age group. M2 cell dominated over M1 cell quantity, and a higher quantity of M2 TAMs was found in skull-base compared with non-skull base tumours. Meningothelial subtypes had a higher quantity of M2 TAMs compared with transitional and atypical ones. Furthermore, the M1/M2-ratio was higher in meningiomas linked to the convexities compared with tumours in the basal. No relations between TAMs and histological WHO grade or prognosis (time to recurrence and overall survival) were found. TAMs were common in our series of meningiomas. However, their infiltration showed no clinicopathological significance. Due to their complex dynamic characteristics and shifting phenotypes, the investigation of these immune cells is demanding. Therefore, the TAMs’ definite role in human meningiomas in relation to clinicopathological parameters and prognosis need to be further investigated.

## Introduction

Meningiomas are mostly benign neoplasms which arise from arachnoid cap cells [1]. Comprising over one-third of primary CNS (central nervous system)-tumours, meningiomas are the most common primary intracranial tumour type in adults with an incidence rate of 9.73 per 100 000, twice as common in women than in men, and rare in children [2]. These tumours occur mainly in the elderly population, and the incidence increases with age [3]. Meningiomas are graded according to the CNS WHO criteria based on histopathological and molecular genetic features [4]. About 80% of meningiomas are considered benign, whilst a limited proportion present with clinical aggressive behaviour with higher relapse rates and invasion of the adjacent brain tissue [3]. Primary treatment of meningiomas are surgery and radiation whereas systemic therapy like immunotherapy is undergoing trials [5–8].

Various immune cells such as lymphocytes, plasma cells, NK-cells, and macrophages/microglia, have been identified in the meningioma tissue. These cells, together with vascular endothelial cells and fibroblasts, constitute a complex tumour microenvironment which promotes and regulates tumour development and growth [9–12]. TAMs (tumour associated macrophages), which are macrophages in tumour tissue, are known to be activated into different phenotypes like classical and alternative macrophages, respectively M1 and M2 phenotypes [13,14]. The macrophage states provide the basis for the established concept of M1 and M2 model. In this model, M1 TAMs are thought to be inflammatory or anti-tumoral and thereby hindering tumour growth/development, and M2 macrophages are believed to be immunosuppressive or pro-tumoral and enhancing tumour growth/development [13,15]. In other tumour types, M2 TAMs have been associated with poorer prognosis and shorter time to recurrence, whilst M1 TAMs have been linked to better prognosis [13,16–18].

In meningiomas, the clinicopathological role of TAMs in relation to tumour growth, development and prognosis has been previously investigated [11,12,19,20]. However, the knowledge of TAMs and M1/M2 cells in relation to meningioma growth needs to be further explored [21]. The aim of this study was therefore to investigate the presence, morphology, and distribution of TAMs in human meningiomas, and relate these findings to histopathology, meningioma subtypes, WHO grade, risk of recurrence, and overall survival.

## Materials and methods

### Collection of patient material and patient specimens

**Fig 1:**
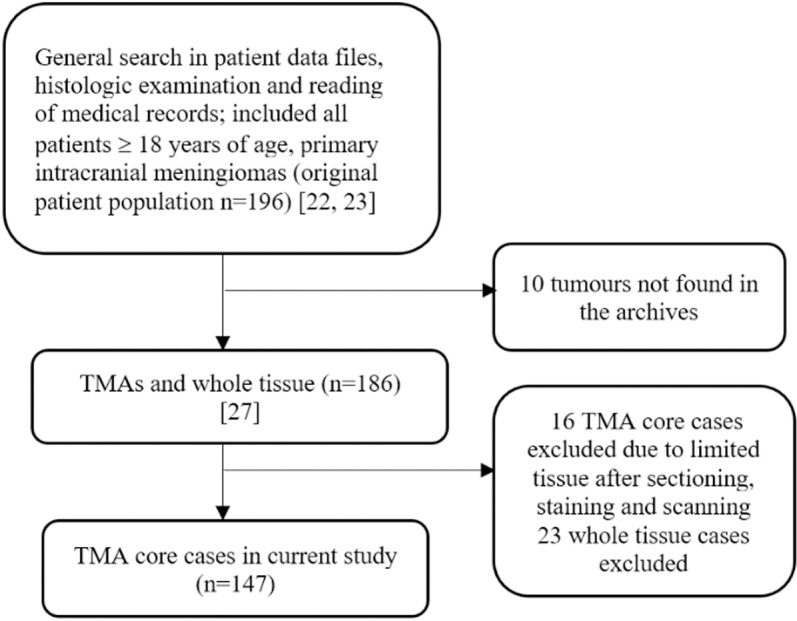
Flow diagram of patient-selection.

The material collection, patient selection, and histopathological evaluation has been described previously [22, 23]. Briefly summarized, a search in the electronic patient data files at Department of Pathology (St. Olav’s University Hospital in Trondheim, Norway) was conducted to find patients operated for primary meningiomas in the time frame between 01.01.1991 to 31.12.2000. The tumour specimens were fixed in formalin and embedded in paraffin shortly after operation. Subsequently, they were stored and later retrieved for review and graded according to the WHO 2016 classification [24]. All patients ≥18 years of age were evaluated for inclusion. A total of 147 meningioma cases were included in the current study with a maximum follow-up time of 18 years. Patients < 18 years of age, non-primary tumours, WHO grade 3, and spinal located meningiomas were excluded from this study (**Fig 1**). Patient data and tumour samples were accessed for research purposes during the year of 2007. In this retrospective study, authors had complete access of information to identify individual participants during and after data collection at all times.

### Clinical data

The clinical data obtained were gender, date and age at surgery, localization of tumour, treatment provided, preoperative symptoms, Simpsons resection grade, WHO performance status, date of recurrence, date and cause of death, and last observation of patients.

Time to recurrence (TTR) as disease recurrence or disease-related death, and overall survival (OS) as time to death irrespective of cause were defined as described in previous protocols [23,25,26]. TTR and OS were calculated by using time of diagnosis as a reference. Survival data was obtained from patient medical files and Norwegian Cause of Death Registry. Surgical notes and radiological analyses (CT and MRI) were used to determine tumour localization. Simpson resection grade was determined and retrospectively assessed by surgical notes.

### Tissue micro arrays (TMAs)

The construction of TMAs has been described in previous studies [27]. Shortly, this has been done by utilizing standardized haematoxylin/eosin sections of tumour specimens to define representative tumour tissue. Three cylinders from each biopsy were cut out, meaning each meningioma case was represented by three TMA extracted cores. Furthermore, the TMA sections were scanned by Olympus VS120S5 scanner at x 20 magnification and prepared for analysis by electronic images by Olympus *OlyVia 3.3*.

### Immunohistochemical procedure for the antibodies Iba1, iNOS and Arginase 1

In this study, three different primary antibodies against macrophages were used: Iba1, iNOS and Arginase 1 (**Table 1**). They were utilized as surrogates for the pan-marker of “all TAMs”, M1 and M2 macrophages, respectively. Formalin fixed, paraffin embedded tissue sections of 4 µm thickness were retrieved from a freezing-unit (stored in the freezer at -20 °C) and put into a heating cabinet for one hour at 60 °C. The tissue sections were deparaffinized by Tissue Clear. Subsequently, ethanol and water were utilized for rehydration. Heat Induced Epitope Retrival (HIER) was performed in a Pre-Treatment Link (DAKO, Glostrup, Denmark) for pre-treatment. The sections were immersed in EnVision FLEX Target Retrieval Solution (High pH 9 for Iba1, and Low pH 6 for iNOS and Arginase). DM 828 (for Iba1) and DM 829 (for iNOS and Arginase) diluted 1:50 with dH_2_O. The temperature was raised to 97 °C for 20 minutes and then cooled to 65 °C. The immunostaining was performed at room temperature by using Dako Autostainer Plus (DAKO). The sections were rinsed with Dako Wash Buffer (DAKO) S3006 1:10 between each step in the immunostaining process. The enzyme blocking was performed with Dako REAL Peroxidase Blocking Solution (DAKO S2023) for ten minutes. The primary antibodies were diluted with Dako REAL Antibody Diluent S2022 and incubated for 30 minutes for Iba1, and overnight at 4 °C for iNOS and Arginase. For Iba1, Rabbit-anti-goat (biotinylated) EO466 from Agilent/Dako dilutions 1:200 in TBS (Tris buffered saline) was incubated for 30 minutes. LSAB-HRP K0675 from Agilent/DAKO ready to use was utilized and then incubated for ten minutes. For iNOS and Arginase, HRP Rabbit/Mouse ENVision Polymer (from Dako REAL Envision DetectionSystem) was incubated for 30 minutes, before sections were rinsed twice. All sections were incubated for ten minutes in DAB+ Chromogen (from Dako REAL EnVision Detection System) for visualization, rinsed twice in dH_2_O, removed from the autostainer, and coloured with contrast Haematoxylin for 30 seconds. The sections were dehydrated and embedded in TissueClear before coverslipping with TissueMount (Sakura). Spleen, lung, and liver were used as positive controls for Iba1, iNOS and Arginase, respectively. In the negative controls the primary antibodies were omitted.

**Table 1:** Key details regarding the antibodies used in this study.

Antibody	Catalogue	Clone	Dilution	Incubation	Producer	Supplier
**Iba1 (“all” TAMs)**	Ab5076	Goat polyclonal Human Iba1	1:500	30 minutes in room temperature	Abcam	Abcam
**iNOS (M1)**	SAB5500152	RbMab_SP16	1:50	Overnight at 4 °C	Sigma Aldrich	Merck
**Arginase 1 (M2)**	93668	RbMab_D4E3M	1:50	Overnight at 4 °C	CST (Cell Signaling Technology)	BioNordika

### Evaluation of specimens

For each case and each antibody, distribution, quantity, and morphology of the TAMs were assessed. The distribution of TAMs was classified as either *“clustered”* or *“dispersed”*. Quantity of TAMs was evaluated as 0 (no positive cells), 1 (sparse cells, <10% of cells in the area of one single TMA core), 2 (moderate of cells, 10–50% of cells in the area of one single TMA core), and 3 (abundant of cells, >50% of cells in the area of one single TMA core). Morphology of TAMs was recorded as *“dominant amoeboid”* (cells having a round shape), or *“dominant ramified”* (cells having protrusions). The TMA sections were scanned using Olympus VS120S5 scanning system, and analyses were consequently conducted by the investigators on electronic images. TMAs with insufficient tumour tissue, meaning < 50% remaining tumour tissue, were excluded from analyses. Quantity of TAMs was analysed by two of the authors (RM and SET), whilst distribution and morphology were assessed by one author (RM). These analyses were made by subjective estimates, whereas discrepancies were brought up for discussion and a consensus was made. The investigators (RM and SET) were blinded for any case-specific clinical data during investigation.

### Digital quantification of Iba-1-, iNOS-, and Arginase-positive cells

Each core of the previously mentioned scanned TMA slides were individually saved as a separate image file. Quantification of Iba1, iNOS, and Arginase positive cells was conducted using the open-source software QuPath [28]. The procedures in QuPath followed the methods outlined in Bankhead et al. with minor modifications to parameters [28]. Representative stained images for each immunostaining were analysed using colour deconvolution. Tissue was detected using the “Create thresholder” tool, with settings including averaging all channels, a threshold of 218, sigma of 2.5, resolution of 2.76 µm/px, and a minimum and maximum area threshold of 5000 µm².

For Iba1-stained TMAs, cell detection was performed with the Stardist extension in QuPath, a convolutional neural network-based method detailed in the paper of Uwe Schmidt et al. and was employed using the “HE heavy augment” pretrained model [29]. The Stardist nucleus detection parameters were set to a requested pixel size of 0.4 µm, threshold of 0.1, and cell expansion of 2.0 µm. Cell detections were filtered to exclude areas smaller than 150 µm² and larger than 3000 µm².

For iNOS and Arginase detection, we utilized QuPath’s built-in watershed cell nucleus detection. This was visually optimized using the following settings: Optical Density (OD) sum; requested pixel size of 0.5 µm; background radius of 8.0 µm; median filter radius of 0.0 µm, sigma of 2.3 µm; minimum and maximum area thresholds of 20 µm² and 400 µm² respectively; a threshold of 0.01; maximum background intensity of 2.0; and cell expansion of 5 µm. Standard feature measurements were supplemented by the “Compute intensity features” for all colour channels/transforms, followed by additional calculations for smoothed object features (spanning 25, 50, and 100 µm). Positive cells were classified using a manually assessed triple-feature simple thresholder, designed to maximize sensitivity and specificity. This involved three measurements: DAB mean cell intensity, nucleus area, and minimal nuclear diameter.

### Statistical analysis

The statistical analyses were performed by using IBM SPSS Statistics version 25. A *p*-value of <0.05 was considered statistically significant. The Spearman’s rank correlation was used to evaluate the correlation between the manual quantity assessment and the digital quantification of TAMs. The manual quantity assessment was provided as a quality assurance for the digital quantification of TAMs. Accordingly, only the digital quantification of TAMs was used for further statistical analyses. The median quantity per mm^2^ was calculated for each case stained with Iba1, Arginase and iNOS and used for all quantity analyses. The Wilcoxon sign rank test was applied to determine if there was a difference in TAMs quantity between M1 (iNOS-stained) and M2 (Arginase-stained) TAMs, and between Total TAMs (Iba1-stained) and M1 (iNOS-stained) + M2 (Arginase-stained). The Mann-Whitney U test was utilized to compare the quantity of TAMs or M1/M2-ratio in the following dichotomic variables: age (Age < 60 years/Age ≥ 60 years), gender (male/female), WHO grade (grade 1/grade 2), and recurrence (recurrence/no recurrence). The Kruskal-Wallis H test was applied to test for association between the quantity of TAMs per mm^2^ or M1/M2-ratio, and independent variables with more than two groups like histologic subtype (transitional, meningothelial, fibrous and atypical), and tumour localization (convexity, skull base, posterior fossa and tentorium, and falcine), followed by pairwise Dunn’s test procedure with a Bonferroni correction for multiple comparisons for preservation of the family-wise error rate if the omnibus test was significant. Histologic subtypes with only one observation were excluded from the Kruskal-Wallis test. The same was done for the intraventricular tumour in the localization analyses. Cox regression analyses with endpoints time to recurrence (TTR) and overall survival (OS) were applied for the continuous covariates TAMs quantity (for each staining) or M1/M2-ratio.

### Ethics

This study was approved by the Regional Committee for Medical and Health Research Ethics Central Norway (project number 4.2006.947). The Committee granted a waiver of informed consent.

## Results

### Clinical and patient data

A total of 147 cases were included in this study, of which 99 cases were WHO grade 1 and 48 cases were WHO grade 2. More women (72.8%) were included than men (27.2%), giving a female:male ratio of 2.7:1. The median age at surgery was 60.0 years (ranging from 27–86 years). The most frequent histologic subtypes were transitional (41.5%), atypical (32.0%), meningothelial (17.0%), and fibrous (5.4%). The tumours were mostly localized to the convexity (49.7%), subsequent by skull base (22.4%) and falcine (15.0%). An overall of 38 (25.9%) cases experienced recurrence, and the recurrence rates were higher for grade 2 tumours (33.3%) compared with grade 1 tumours (22.2%). An in-depth overview of the clinicopathological data is displayed in **Table 2**.

**Table 2:** Clinical data^a^.

Category	Total	WHO grade 1	WHO grade 2
**No. of cases**	147 (100)	99 (67.3)	48 (32.7)
**Age, ** *median, mean, (range)*	60.0, 60.2, (27-86)	59.4, 59.0, (27-84)	61.9, 65.0, (30-86)
**Gender**			
*Women*	107 (72.8)	74 (74.7)	33 (68.8)
*Men*	40 (27.2)	25 (25.3)	15 (31.2)
**Histologic subtype**			
*Transitional*	61 (41.5)	61 (61.6)	0 (0.0)
*Meningothelial*	25 (17.0)	25 (25.3)	0 (0.0)
*Fibrous*	8 (5.4)	8 (8.1)	0 (0.0)
*Angiomatous*	1 (0.7)	1 (1.0)	0 (0.0)
*Psammomatous*	1 (0.7)	1 (1.0)	0 (0.0)
*Secretory*	1 (0.7)	1 (1.0)	0 (0.0)
*Lymphoplasmacyte-rich*	1 (0.7)	1 (1.0)	0 (0.0)
*Metaplastic*	1 (0.7)	1 (1.0)	0 (0.0)
*Atypical*	47 (32.0)	0 (0.0)	47 (97.9)
*Clear cell*	1 (0.7)	0 (0.0)	1 (2.1)
**Tumour localization**			
*Convexity*	73 (49.7)	44 (44.5)	29 (60.4)
*Skull base*	33 (22.4)	28 (28.3)	5 (10.4)
*Posterior fossa and tentorium*	18 (12.2)	14 (14.1)	4 (8.3)
*Falcine*	22 (15.0)	13 (13.1)	9 (18.8)
*Intraventricular*	1 (0.7)	0 (0.0)	1 (2.1)
**Recurrence**			
*Yes*	38 (25.9)	22 (22.2)	16 (33.3)
*No*	109 (74.1)	77 (77.8)	32 (66.7)

^a^Values are given as number (%) unless otherwise specified.

### Morphology of TAMs

**Fig 2:**
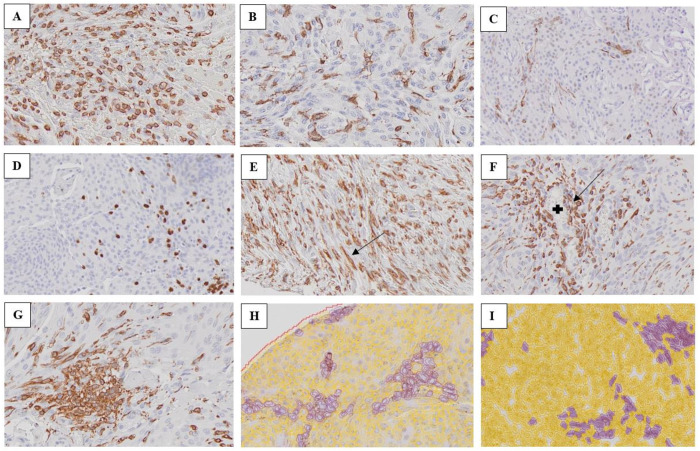
Meningioma tissue illustrating A; abundant quantity of TAMs stained with Iba1 with dominant amoeboid morphology originally in a dispersed distribution (WHO grade 1), B; moderate quantity of TAMs stained with Iba1 with ramified cells expressing their processes in a dispersed distribution (WHO grade 1), C; sparse quantity of iNOS-stained TAMs with ramified cells in a dispersed distribution (WHO grade 1), D; sparse quantity of Arginase-stained TAMs with dominantly amoeboid/round shaped cells in a dispersed distribution (WHO grade 1), E; arrow pointing on Iba1-stained TAM with rod-shaped morphology (WHO grade 1), F; arrow pointing on Iba1-stained TAMs around a blood vessel marked with a plus sign (WHO grade 1), G; clustered distribution of Iba1-stained TAMs (WHO grade 1), H; iNOS-stained TAMs during the annotation process in regard to the digital quantification of macrophages (WHO grade 1) I: iNOS- stained TAMs during annotation process in regard to the digital quantification (WHO grade 2). (Original magnification x 20).

The morphological appearances of TAMs were recorded as amoeboid or ramified (Figs 2A and B, respectively). Occasionally, round shaped, or rod-shaped cells were observed as well (Figs 2D and E). No TAMs displayed a ramified morphology when the antibody Arginase was used. Figs 2H and 2I represent parts of the annotation process in iNOS (M1)-stained TAMs in relation to the digital assessment of these cells. See Table 3 for frequencies of the morphology.

**Table 3:** Results from the immunohistochemical investigation^b^.

Immunohistochemical categories	Total	WHO grade 1	WHO grade 2
*Iba1*			
Quantity of TAMs per mm^2^ *median, (minimum, maximum)*	1524.9 (18, 6372)	1514.7 (18.4, 6372.5)	1624.9 (151.9, 5388.1)
Dispersed/Clustered distribution	138 (93.9)/9 (6.1)	96 (97.0)/ 3 (3.0)	42 (87.5)/ 6 (12.5)
Amoeboid/Ramified morphology	53 (36.1)/94 (63.9)	36 (36.4)/63 (63.6)	17 (35.4)/31 (64.6)
*i* ***NOS***			
Quantity of TAMs per mm^2^ *(median, minimum, maximum)*	2.2 (0, 669)	2.2 (0, 669)	2.2 (0, 185.4)
Dispersed/Clustered distribution	20 (62.5)/12 (37.5)	9 (56.3)/7 (43.8)	11 (68.8)/ 5 (31.3)
Amoeboid/Ramified morphology	11 (34.4)/21 (65.6)	6 (31.6)/13 (68.4)	5 (38.5)/8 (61.5)
*Arginase*			
Quantity of TAMs per mm^2^ *(median, minimum, maximum)*	3.8 (0, 224)	3.8 (0, 224.5)	3.9 (0.6, 40)
Dispersed/Clustered distribution	78 (84.8)/ 14 (15.2)	48 (85.7)/ 8 (14.3)	30 (83.3)/ 6 (16.7)
Amoeboid/Ramified morphology	92 (100)/ 0 (0.0)	56 (100)/ 0 (0.0)	36 (100)/ 0 (0.0)

^b^Values are given as number (%) unless otherwise specified

### Distribution of TAMs

The TAMs appeared both in dispersed and clustered distributions (Figs 2B and G), scattered in tumour tissue as well as around and in proximity to blood vessels (Fig 2F). However, more cases showed a dispersed distribution of Iba1-stained TAMs, both in total and within WHO grade 1 and 2 (Figs 2A and B). See Table 3 for frequencies of the distribution.

### Digital and manual assessment of the amounts of TAMs

There was a significant positive correlation between manual quantity assessments and their respective digital quantifications of TAMs: Iba1 (r=0.821, *p* < 0.001), iNOS (r= 0.508, *p* < 0.001), and Arginase (r= 0.610, *p* < 0.001). Accordingly, all further analyses were based on digital TAMs quantification.

### Quantity of TAMs

All cases showed Iba1-immunoreactivity for TAMs, while 32 cases were positive for iNOS- and 92 cases for Arginase (Figs 2A, B, E, F and G for Iba1- stained TAMs, and Figs 2C and D for iNOS- and Arginase1-stained TAMs, respectively). Furthermore, there was a significantly higher amount of M2 macrophages compared with M1 TAMs in the tumours overall (Z = -4.122, *p*-value = < 0.001). Total amount of TAMs (Iba1) was also significantly higher than M1 (iNOS) + M2 (Arginase) cells (Z= -10,466, *p*-value = <0.001), which can also be comprehended by the difference of medians for the respective quantities of cells shown in Table 3.

Further, there was also a significant higher quantity of Iba1-stained TAMs in the age group ≥ 60 years compared with the age group < 60 years (with median quantity 1953.2 and 1143.5 per mm^2^, respectively (p-value <0.001)). For the other dichotomic variables including gender, WHO grade, and recurrence status, we found no significant differences in median quantity of macrophages (Table 4).

**Table 4:** p-values extracted from the two-tailed exact Mann-Whitney U test.

Antibody	Mann-Whitney U	*n*	Quantity	
**Categorical variables**	**Median (range)**	*p* **-value**
**Iba1**	Age < 60 years Age ≥ 60 years	71 76	1143.5 (18.4–5088.2) 1953.2 (27.9–6372.5)	**< 0.001**
	Male Female	40 107	1592.6 (18.4–5388.1) 1199.6 (151.9–6372.5)	0.408
	WHO grade 1 WHO grade 2	99 48	1514.7 (18.4–6372.5) 1624.9 (151.9–5388.1)	0.381
	Recurrence No recurrence	38 109	1630.9 (18.4–4184.0) 1514.9 (27.9–6372.5)	0.940
**iNOS**	Age < 60 years Age ≥ 60 years	71 76	2.4 (0.0 -669.0) 1.9 (0.0 - 84.7)	0.182
	Male Female	40 107	2.1 (0.0–669.0) 2.35 (0.0–403.9)	0.333
	WHO grade 1 WHO grade 2	99 48	2.2 (0.0–669.0) 2.2 (0.0–185.4)	0.786
	Recurrence No recurrence	38 109	2.2 (0.0–140.3) 2.1 (0.0–669.0)	0.988
**Arginase**	Age < 60 years Age ≥ 60 years	71 76	4.8 (0.6–224.5) 3.3 (0.6–40.0)	0.086
	Male Female	40 107	3.8 (0.6–152.7) 4.0 (0.0–224.5)	0.794
	WHO grade 1 WHO grade 2	99 48	3.8 (0.0–224.5) 3.9 (0.6–40.0)	0.707
	Recurrence No recurrence	38 109	6.3 (0.6–149.2) 3.7 (0.0–224.5)	0.169
**M1/M2-ratio**	Age < 60 years Age ≥ 60 years	71 76	0.53 (0.01–176.05) 0.45 (0.02–81.00)	0.801
	Male Female	40 107	0.44 (0.05–81.00) 0.59 (0.01–176.05)	0.416
	WHO grade 1 WHO grade 2	99 48	0,44 (0.01–176.05) 0.60 (0.05–40.33)	0.413
	Recurrence No recurrence	38 109	0.34 (0.03–22.27) 0.53 (0.01–176.05)	0.074

Significant results are represented in bold

The pairwise comparison for Arginase-stained TAMs showed statistically significant differences in median quantity score of macrophages per mm^2^ between the falcine (3.0) and skull base (6.9) (*p*-value = 0.045), and between the convexity (2.8) and skull base (6.9) (*p*-value = 0.004). This indicates a higher quantity of M2 cells in the skull-base tumours compared with non-skull base tumours. The other pairwise comparisons did not show significantly differences in median TAMs quantity; falcine – convexity (p-value = 1.000), falcine – posterior fossa/tentorium (p-value = 0.392), convexity - posterior fossa/tentorium (p-value = 0.182), posterior fossa/tentorium – basal (p-value = 1.000) (Table 5).

**Table 5:** Digital quantity scoring (per mm^2^) of TAMs or M1/M2-ratio related to tumour localization and histologic subtype.

Kruskal-Wallis H test		Iba1		iNOS		Arginase		M1/M2-ratio	
**Independent variables**		**Median** **(Q1, Q3)**	*p***-value** *(*T*, Df)*	**Median** **(Q1, Q3)**	*p***-value** *(*T*, Df)*	**Median** **(Q1, Q3)**	*p***-value** *(*T*, Df)*	**Median (Q1, Q3)**	*p***-value** *(*T*, Df)*
**Tumour localization**	*n*								
Convexity	73	1711.1 (903.0, 2559.8)	0.075 (X^2^ (3) = 6.9)	2.00 (0.7, 4.4)	0.799 (X^2^ (3) = 1.0)	2.8 (1.8, 6.7)	**0.002** (X^2^ (3) = 15.2)	0.53 (0.26, 1.65)	**0.045** (X^2^ (3) = 8.1)
Skull base	33	1155.0 (605.4, 1760.0)		2.2 (0.8, 6.9)		6.9 (3.4, 25.7)		0.25 (0.10, 0.72)	
Posterior fossa and tentorium	18	1429.8 (923.9, 2707.1)		2.2 (1.4, 6.5)		8.4 (1.7, 23.8)		0.44 (0.16, 1.00)	
Falcine	22	1739.1 (959.5, 2745.0)		2.85 (0.7, 6.1)		3.0 (1.8, 7.2)		0.73 (0.23, 1.13)	
**Histologic subtype**									
Transitional	61	1607.3 (1025.2, 2614.1)	0.502 (X^2^ (3) = 2.4)	1.7 (0.7, 4.4)	0.459 (X^2^ (3) = 2.6)	3.3 (1.5, 6.9)	**0.005** (X^2^ (3) = 12.8)	0.53 (0.23, 1.35)	0.139 (X^2^ (3) = 5.5)
Meningothelial	25	1334.6 (867.2, 1773.2)		3.1 (1.4, 5.6)		9.9 (3.8, 15.7)		0.28 (0.12, 0.53)	
Fibrous	8	1480.0 (577.35, 2174.6)		1.95 (0.35, 4.1)		3.9 (1.45, 12.5)		0.32 (0.11, 0.86)	
Atypical	47	1711.1 (988.55, 2481.45)		2.2 (0.8, 6.15)		3.7 (2.15, 7.65)		0.61 (0.25, 1.5)	

Significant results are represented in bold

Q 1: 25^th^ percentile

Q 3: 75^th^ percentile

T : test statistics

Df: degree of freedom

We found statistically significant differences in median quantity score of Arginase-stained TAMs per mm^2^ between the transitional (3.3) and meningothelial (9.9) (*p*-value = 0.002), and between the meningothelial (9.9) and atypical (3.7) (*p*-value = 0.038) histologic subtype-groups. This indicated a higher quantity of M2 cells in meningothelial subtype compared with transitional and atypical subtypes. The other pairwise comparisons did not show significantly differences in median TAMs quantity: transitional – fibrous (p-value = 1.000), transitional – atypical (p-value = 1.000), fibrous - atypical (p-value = 1.000), and fibrous – meningothelial (p-value = 0.514).

Further, we did not find any statistically significant difference of median quantity score in relation to tumour localization or histologic subtypes in respect of Iba1- and iNOS-stained TAMs (**Table 5**).

### M1/M2-ratio of TAMs

We calculated an M1/M2-ratio for each case included in the study, and the median ratio was found to be 0.45. There was no difference in median M1/M2-ratio between age-groups, gender, WHO-grades, recurrence-status, or between histologic subtypes (Tables 4 and 5). However, the pairwise comparison for M1/M2-ratio showed statistically significant differences in median M1/M2-ratio in respect of tumour localization groups (*p*-value = 0.045, X^2^(3) = 8.070) between the convexity (0.53) and the basal (0.25) (p-value = 0.035) indicating a lower M1/M2-ratio in skull-base tumours. The other pairwise comparisons did not show significantly differences in median M1/M2-ratio; basal - posterior fossa/tentorium (p-value = 1.000), basal – falcine (p-value = 0.335), posterior fossa/tentorium - falcine (p-value = 1.000), posterior fossa/tentorium – convexity (p-value = 1.000), falcine – convexity (p-value = 1.000).

### TAMs quantity or M1/M2-ratio and prognosis in meningiomas

In the univariable survival analyses, the TAM quantity in respect of Iba1, iNOS and Arginase, or M1/M2-ratio were not shown to have statistically significant associations with recurrence or disease-related death (TTR), nor overall survival (OS) (Table 6).

**Table 6:** Associations between TAMs quantity or M1/M2-ratio, and TTR or OS from univariate Cox regression analyses.

Variable of interest	Measure	TTR (n=52)	OS (n=59)
Iba1	HR (CI) (*p*-value)	1.000 (1.000–1.000) (0.794)	1.000 (1.000–1.000) (0.978)
iNOS	HR (CI) (*p*-value)	0.997 (0.989 -1.005) (0.423)	0.998 (0.993–1.004) (0.491)
Arginase	HR (CI) (*p*-value)	1.002 (0.994–1.011) (0.639)	0.986 (0.969–1.003) (0.114)
M1/M2-ratio	HR (CI) (*p*-value)	0.982 (0.940–1.024) (0.392)	0.996 (0.977–1.015) (0.656)

Significant results are highlighted in bold

HR: Hazard ratio

CI: 95% confidence interval

TTR: time to recurrence or disease-related death

OS: overall survival

## Discussion

To our knowledge, the current research on TAMs’ clinical role in human meningiomas is sparse. In this study, we found that TAMs are commonly present in meningiomas. The number of TAMs were observed to increase with patient age, and a higher amount of M2 TAMs were found in skull-base compared with non-skull base tumours. We did not find that TAMs quantity or M1/M2 ratio were associated with gender, tumour grade, or prognosis.

### TAMs in meningiomas

Iba1 is a non-specific pan-marker for microglia/macrophages [15,30]. This antibody was utilized to define the extent of total or “all” TAMs in the tumour tissue of each meningioma case to assess the distribution and morphological appearances. The latter may reflect their activation state; ramified appearances are linked to a steady or resting state, whilst amoeboid morphology is considered to reflect an active phase [30, 31]. We observed that the TAMs appeared as ramified, amoeboid, and occasionally also rod- or round shaped, which is in accordance with Asai et al [11]. They were predominantly randomly dispersed in the meningioma tissue and occasionally in clusters. The random distribution of TAMs is somewhat different from that in glioblastomas, where TAMs seem to appear in more specific locations such as in central tumour areas, necrotic areas, perivascular, and in the infiltration zones [30].

Patient age seems to influence the quantity of TAMs in meningiomas, as the number of Iba1-stained TAMs was significantly higher in the older compared to the younger age group. Meningiomas are often slow-growing neoplasms most common in adulthood with a median age of 67 years, so one must speculate whether this facilitates tumour growth because of reduced activity of TAMs in the elderly [2,24]. For instance, it is reported that macrophages from older mice show lower antioxidant defence, higher oxidizing products and increased levels of lipofuscin [32]. Furthermore, it is reported higher number of TAMs in aged patients with colorectal cancer as age is linked to chronic low-grade inflammation, where an aged tumour microenvironment affects both tumour progression and therapy outcomes [33–37]. Thus, this age-related oxidative stress in macrophages and chronic low-grade inflammation may be a part of the tumorigenesis of human meningiomas. As there is possibly reduced activity of the TAMs in the elderly, this is an important aspect to be considered in immunotherapy for this patient group.

Further, we found that gender was not associated with TAMs’ quantity or M1/M2-ratio. In the study of Ding et al, the investigators did not find any relationship between CD68+ (another pan-macrophage marker) cell subsets and gender as well [38].

### M1 and M2 TAMs

We used immunohistochemistry with antibodies against iNOS and Arginase 1 to define M1 and M2 cells, respectively. Both subsets of TAMs were present in our set of meningiomas. However, TAMs have been suggested to be in various phenotypes in the tumour microenvironment acting in a dynamic manner, by far more complex than the *in vitro* established M1 and M2 cell subsets [10,13,17,39]. M2 cells can also be subgrouped into M2a, M2b, and M2c types, or TAMs classified into angiogenic, immunosuppressive, invasive, metastasis-associated, perivascular, and activated macrophages based on their extensive activation states [17,39]. Some macrophages even present with hybrid M1/M2 phenotypes [13]. Hence, TAMs may hold a wide range of extensive functional phenotypes, where the polarization of activation states depends on the local milieu around these immune cells as well as to cytokine exposure [10,17].

Further, we found a significantly higher amount of M2 in comparison to M1 TAMs, which is in accordance with Proctor et al [19]. This is somewhat unexpected as meningiomas are mostly slow-growing neoplasms with a benign clinical behaviour [3,4,24]. On the other hand, this finding could also be considered as expected as M2 cells have been shown to promote tumour growth. Accordingly, this stands out as a potential target for immunotherapeutic approaches by switching TAMs from M2 to M1 phenotypes [40].

The meninges of the convexity have been shown to originate from the neural crest and meninges of the skull from mesoderm [41, 42]. Furthermore, the embryonic origin is also associated with the fact that WHO grade 1 meningiomas, meningothelial subtype and non-*NF2* tumours are mainly located at the skull base, whereas *NF2* mutations, WHO grade 2/3 tumours and fibrous subtype are more commonly found in the convexities [41–43]. Based on these premises, as well as that the tumours in the skull-base in general are of lower tumour-grade and have better prognosis than the tumours of the convexities, we expected to find a higher number of antitumoral M1 cells as well as a high M1/M2-ratio in skull base meningiomas [42]. However, we found that the number of pro-tumoral M2 TAMs was significantly higher in skull base meningiomas compared with those located in the convexities, as well as a higher M1/M2-ratio in the convexities compared with the skull base. We have no obvious explanation for this discrepancy. One may speculate that the anatomical locations of meningiomas possibly affect the TAMs infiltration due to multiple factors like slow/no growth of tumours in skull-base, patient characteristics such as age, tumour grade, chemoattractant proteins, and/or mutations and cytogenetic changes in a complex interplay in the tumour microenvironment and its surroundings [9,44–47]. For instance, NF2-status may influence the infiltration of various inflammatory cells in the meningioma tissue [47–49]. Further, in the study of Wach et al, they present a higher macrophage infiltration intracranially in comparison with spinal meningiomas [50]. Thus, the circumstances regarding TAMs infiltration in the various histologic subtypes and locations requires further investigation.

The literature is ambiguous whether inflammatory cell infiltration in human tumours is linked to improved prognosis for patients or not [51]. In the present study, we have shown that TAMs quantity and M1/M2-ratio were not associated with recurrence-status, WHO grade, survival and prognosis. This contrasts with the study of Proctor et al, in which they found a significantly decreased M1/M2-ratio in WHO grade 2 compared to WHO grade 1 tumours and more than a two-fold difference in this ratio between primary and recurrent tumours [19]. Rutland et al also found that aggregated macrophages were associated with higher tumour grade [48]. These discrepancies may be due to the dynamic shifting phenotypes of TAMs, the use of different antibodies, or registration techniques [10]. Compared to previous studies, it could also be due to the differences in study design, and the number of cases or patients included. Since the M1/M2-ratio has been identified as important in several studies and the prognostic value is unclear, this issue should be further explored.

We found that the quantity of immunoreactive Iba1 TAMs was not equal to immunoreactive iNOS M1 and Arginase M2 TAMs (TotTAMs≠M1+M2). This could be due to the choice of antibodies or registration techniques in the present study. In that regard, we could have considered applying other antibodies reactive against M1 and M2 phenotypes. As the M1/M2 model is utilized in many reports on human tumours, including meningiomas, and most studies make a strict distinction between M1 and M2 phenotypes, a more standardized procedure to define these phenotypes should be considered [13,15,19,39]. The M1/M2 model is a simplified theoretical moment of a time model or snapshot based on *in vitro* observations. Thus, it differs from the dynamic conditions *in vivo* in the tumour microenvironment with its immune cells and plethora of cytokines/chemokines, non-immune cells, neoplastic cells, cancer stem cells, and extracellular matrix components, which continuously modulates the activation status of TAMs [10,13,17,52]. This dynamic shift of phenotypes may constitute a source of error in the immunohistochemical determination of the various phenotypes and calculation of M1/M2 ratios [10]. Accordingly, studies on the M1/M2 model are encumbered with many uncertainties. On the other hand, the concept of a pro-tumoral (M2) and anti-tumoral (M1) subsets of macrophages may be beneficial in future anti-cancer treatment options. For example, the possibility of reprogramming TAMs from the M2 to M1 phenotype may be of interest, as well as finding compounds that will inhibit macrophage differentiation into M2 cells [17,53–55].

### Strengths and limitations

The strength of this study is the large number of patients included with substantial clinical data and a long follow-up period. In addition, all patients are from one treatment centre with a population-based referral. The digital approach ensures an objective quantification of TAMs, and the use of TMAs contributes to the inclusion of relatively homogeneous tumour tissue, avoiding areas of necrosis and bleeding.

Limitations are the retrospective nature of this study, the inherent challenges of immunohistochemistry including possible reduced sensitivity and specificity of the TAMs. Other limitations to mention are in relation to the use of TMAs which may be under the influence of selection bias as the sample selection for the TMAs are limited in reflecting the whole tumour tissue and the TMA cores are selected based on criteria in a non-random fashion. In the current study, there is also a limitation in the fact that it does not include longitudinal data to assess the changes in TAM populations over time, something that could have been interesting to investigate. Since TAMs possess characteristics such as plasticity, investigation of longitudinal data with the exploration of M1 and M2 cells would yield beneficial knowledge on the topic of shifting phenotypes over time in the same meningioma patients. Further, the manual and digital assessment of the TAMs holds also limitations regarding the subjective opinion of the investigator, and possible reduced specificity of the TAMs which is reliable on adequately trained software to distinguish TAMs from other cells in the tumour tissue, respectively.

## Conclusion

TAMs are common in human meningiomas with both M1 and M2 phenotypes present. However, their infiltration in our set of meningiomas were shown to have no significant relation to age, gender, WHO grade, tumour recurrence, prognosis or M1/M2-ratio. To date, there are few studies on TAMs in meningiomas, and further investigations are needed to elaborate their role in these tumours, especially as far as immunotherapy is concerned.
